# Comparison of high-frequency ultrasound transducers for microvascular localization microscopy in the mouse brain

**DOI:** 10.1162/IMAG.a.151

**Published:** 2025-09-15

**Authors:** Matthew R. Lowerison, Yike Wang, Bing-Ze Lin, Zhe Huang, Dongliang Yan, YiRang Shin, Pengfei Song

**Affiliations:** Department of Electrical and Computer Engineering, University of Illinois Urbana–Champaign, Urbana, IL, United States; Beckman Institute for Advanced Science and Technology, University of Illinois Urbana–Champaign, Urbana, IL, United States; Department of Biomedical Engineering, Duke University, Durham, NC, United States; Neuroscience Program at the University of Illinois Urbana–Champaign, Urbana, IL, United States

**Keywords:** high-frequency ultrasound, super-resolution, ultrasound localization microscopy, neurovascular imaging, microvasculature

## Abstract

Ultrasound localization microscopy is a super-resolution vascular imaging technique which has garnered substantial interest as a tool for small animal neuroimaging, neuroscience research, and the characterization of vascular pathologies. In the context of small animal neurovascular imaging, we posit that increasing the ultrasound imaging frequency is a straightforward approach to enable higher concentrations of microbubble contrast agents, thus increasing the likelihood of microvascular mapping and decreasing the imaging duration. To test this hypothesis, we compared ULM imaging resolution of mouse brain vasculature for three transducers with different center transmit frequencies (15 MHz, 23 MHz, and 31 MHz) under conditions of low and high MB concentration. We demonstrate that higher frequency imaging resulted in more efficient microbubble localization due to a smaller microbubble point-spread function that is easier to localize, and which can achieve a higher localizable concentration within the same unit volume of tissue. We found that increasing the imaging frequency had a minor impact on ULM spatial resolution, as measured by Fourier ring correlation, under the low MB concentration case, but a substantial impact in the high MB concentration case. High-frequency ULM yielded a spatial resolution of 6.9 μm, as measured by Fourier ring correlation, throughout the entire depth of the brain. This highlights the potential of this technology as a highly relevant tool for neuroimaging research, which has substantial implications for neuroscientists investigating microvascular function in disease states, regulation, and brain development.

## 1 Introduction

Ultrasound localization microscopy (ULM) is a super-resolution imaging technology which has rapidly gained traction for small animal cerebrovascular imaging (Couture et al., 2018; Song et al., 2023). First proposed more than a decade ago (Couture et al., 2011; Siepmann et al., 2011), ULM exploits intravascular microbubble (MB) contrast agents to reconstruct vasculature at an imaging resolution below the diffraction limit (Christensen-Jeffries et al., 2015; Errico et al., 2015). The refinement and optimization of ULM processing is an ongoing and rapidly developing area of research; but generally, ULM relies on the sub-wavelength localization of isolated MBs. This necessitates spatially sparse MBs in an imaging frame to avoid interference from overlapping or adjacent MBs. These MB localizations are then tracked frame-to-frame to generate MB trajectories which provide super-resolution vascularity maps as well as physiological indices of blood flow dynamics, including velocity (Christensen-Jeffries et al., 2015; Errico et al., 2015), tortuosity (Shelton et al., 2015), and pulsatility (Bourquin et al., 2022).

Numerous research groups have been actively developing and applying ULM to neuroimaging and to a wide range of cerebrovascular pathologies. There has been a plethora of demonstrations of the technology in imaging neuroscience, including investigation into neurovascular coupling (Renaudin et al., 2022), the effect of aging on cerebrovasculature (Lowerison et al., 2022), neurodegenerative diseases such as Alzheimer’s disease (Lin et al., 2024; Lowerison et al., 2024), hydrocephaly (Zhang et al., 2022), and stroke in rodent models (Chavignon et al., 2022) and in the clinic (Demené et al., 2021). Ultimately, the end goal of ULM research is to provide meaningful physiological indices of microvascular function that are either not possible or not pragmatic with other imaging technologies. However, a consequence of the strategy of relying on sparse MB concentrations is that the technology requires long imaging durations to gradually populate a vascular map (Christensen-Jeffries et al., 2019; Hingot et al., 2019; Lowerison et al., 2020). This, in turn, is a substantial barrier to the technique, as it necessitates expensive data acquisition practices and requires minimal tissue motion.

A potential remedy to rare MB events in small vessels is to increase the concentration of MBs, resulting in more traversal events and therefore a higher probability of reconstructing microvascular flow. However, this is at odds with the requirement for spatially sparse MB signals. This problem has motivated several technical developments to extract meaningful MB localization data from overlapping signals in the computational/post-processing domain. These include Fourier-domain filters to synthetically separate MBs (Huang et al., 2020), compressed sensing (Kim et al., 2021), sparse image recovery (Bar-Zion et al., 2018; Yan et al., 2022), and several demonstrations of deep-learning to extract MB locations (Chen et al., 2022; Shin et al., 2024; van Sloun et al., 2021). These techniques offer powerful and data-driven approaches to mitigate the high-density localization problem, which may be combined with strategies for better localization in the data acquisition/imaging domain.

We posit that one such strategy for better MB localization in the imaging domain is increasing the ultrasound imaging frequency. It should be noted that the imaging penetration depth is proportional to the wavelength, thus the maximum imaging depth will be reduced at higher frequencies, which is a trade-off that needs to be carefully considered. For small animal neuroimaging there is ample room to explore high(er) imaging frequencies (e.g., 30 MHz) due to the relatively shallow imaging target and the strong acoustic backscatter of MB contrast. This provides a straightforward avenue for enabling accurate localization at high(er) concentrations of MBs. MBs are designed to be <5 μm in diameter to enable vascular passage without risk of embolism. Thus, MBs represent true point scatterers for most physiologically relevant imaging frequencies and should closely resemble the PSF of the ultrasound imaging system. As the imaging frequency increases, the physical extent of the PSF will narrow, and more MBs can occupy the same unit volume while remaining localizable (Belgharbi et al., 2023; Christensen-Jeffries et al., 2019).

To this end, we investigated the use of a high-frequency ultrasound imaging transducer (center transmit of 31 MHz) for ULM imaging of mouse brain vasculature. We compared the ULM imaging performance with two lower frequency transducers (15 MHz and 23 MHz center transmit) under conditions of low and high MB concentrations. All other imaging acquisition and experimental parameters were kept as consistent as possible to minimize confounding sources of reconstruction variance. We demonstrate that a higher imaging frequency enables higher MB concentrations, potentially allowing for reduced data acquisition times. The high-frequency imaging was further tested on a Vantage NXT system which provides a higher sampling rate that alleviates the issue of interleaved sampling on the Vantage 256 system. This provides a higher attainable imaging framerate which is beneficial for the ULM tracking step. This dataset was able to achieve a resolution of 6.9 ± 0.2 μm, as measured by Fourier ring correlation, for the entire depth of the brain. This has substantial implications for neuroscience, as microvascular imaging is a crucial tool for understanding neurodegenerative diseases, brain development, and neuro-regulation.

## 2 Methods

### 2.1 Ethics statement

All procedures conducted on mice presented in this article were approved by the Institutional Animal Care and Use Committee (IACUC) at the University of Illinois Urbana-Champaign (protocol #22033). All presented experiments were conducted in accordance with the IACUC guidelines. Mice were housed in an animal care facility approved by the Association for Assessment and Accreditation of Laboratory Animal Care. Every attempt was made to minimize the number of animals used and to reduce suffering at all stages of the study.

### 2.2 Animal model

**Fig. 1.**
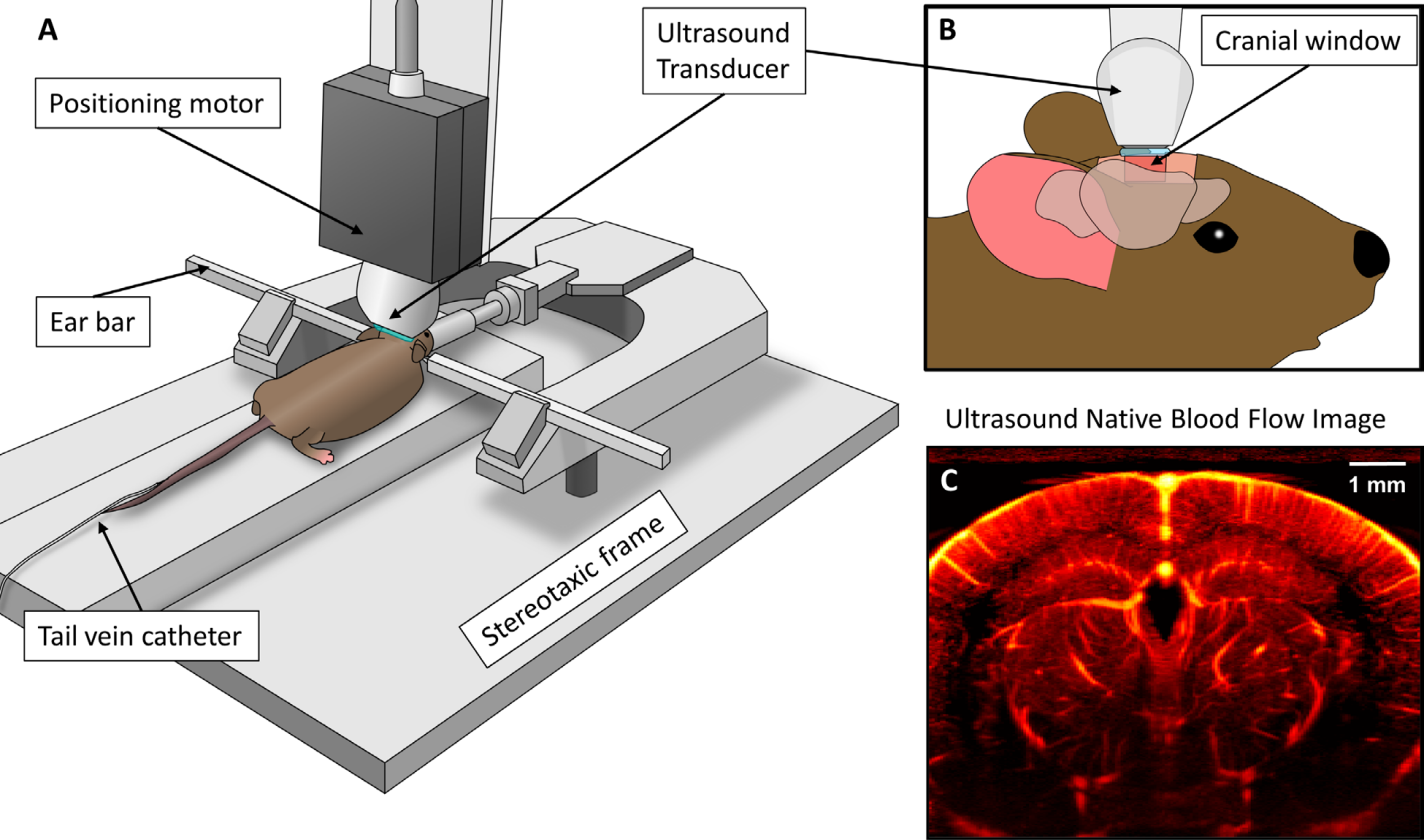
(A) Schematic of experimental setup, including stereotaxic imaging frame and positioning motor for placement of ultrasound transducer. (B) Side view of ultrasound imaging through rectangular cranial window opened in the mouse skull, with the imaging plane positioned at approximately 2 mm caudal to bregma. (C) Example ultrasound power Doppler blood flow image, demonstrating select anatomies of interest, such as the hippocampus, thalamus, and cortical regions.

Mouse anesthesia was initialized using a gas induction chamber supplying 4% isoflurane mixed with oxygen. Anesthesia was maintained via a nose cone supplying 2% isoflurane for all procedures. Once anesthetized, the mouse was transferred to a stereotaxic imaging stage (Model 900-U, David Kopf Instruments, Tujunga, CA, USA), and its head was secured in place via ear bars. A cranial window spanning from bregma to lambda was opened in the skull using a rotary tool (Model K1070, Foredom, Bethel, CT, USA) to expose the bilateral expanse of the cerebral cortex. Animal body temperature was maintained at 37 °C using a temperature controller (TCAT-2, Physitemp Instruments, Clifton, NJ, USA). A schematic of this imaging setup is demonstrated in Figure 1.

A 29-gauge needle-tip catheter (CA-0099EO, Braintree Scientific, MA, USA) was inserted into the tail vein of the mouse, and vessel patency was confirmed with a 50 μL saline bolus. Commercially available MB contrast agent (Lumason^®^, Bracco, Milan, Italy) was activated following the supplier’s direction, yielding a solution with approximately 3.5 x 10^8^ MBs/mL. This MB solution was infused through the tail vein catheter at a rate of 20μL/min using a programmable syringe pump (NE-300, New Era Pump Systems Inc., Farmingdale, NY, USA). The MB solution was mixed every 3 minutes using a custom-built magnetic stirrer to keep the MB concentration consistent throughout the experiment. High MB concentration data acquisitions were accomplished by increasing the flow rate to 40μL/min.

### 2.3 Ultrasound data acquisition

The majority of ultrasound data collection was performed using a Vantage 256 system (Verasonics Inc., Kirkland, WA, USA), with an additional high-frequency dataset acquired using a Vantage 256 NXT system (Verasonics Inc., Kirkland, WA, USA). Three different linear array ultrasound transducers were used for imaging: an L22-14vX (Verasonics Inc., Kirkland, WA, USA), an L35-16vX (Verasonics Inc., Kirkland, WA, USA), and an MS-550S (FUJIFILM VisualSonics, Toronto, Canada). Details about these arrays can be found in the following table (Table 1), where the elevational beam width was estimated using the methods described in Section 2.6. All imaging was performed with 9-angle plane-wave compounding (-4 to 4, 1-degree increment), a 1000 Hz post-compounding frame rate, and a one-cycle transmit pulse (duty cycle less than 0.8% for all sequences). The transmit voltage for each transducer was adjusted to achieve a target MI of 0.05 (5 volt for the L22-14vX, 6 volt for the L35-16vX, and 8 volt for the MS-550S) as measured by a capsule hydrophone (HGL-0200, Onda Corporation, Sunnyvale, CA, USA). In all cases, the TGC was maxed for each experiment. Data were acquired and saved as RF super-frames corresponding to ensembles of 1000 post-compounded IQ frames per acquisition. A 2-1 interleaved sampling strategy (‘NS200BWI’) was employed to effectively double the Vantage’s 62.5 MHz ADC receive sampling rate for the L35-16vX and MS-550S acquisitions. High-frequency imaging was also performed with a Vantage NXT system (Verasonics Inc., Kirkland, WA, USA), where no interleaved sampling was necessary because of the higher system sampling frequency (125 MHz).

**Table 1.** Transducer specifications for L22-14vX, L35-16vX, and MS550-S.

Transducer	L22-14vX	L35-16vX	MS-550S
Transmit frequency [MHz]	15	23	31
Element count [N]	128	128	256
Pitch [mm]	0.1	0.07	0.055
Lateral aperture size [mm]	12.8	8.9	14.08
Elevational beam width [mm]	0.90 ± 0.21	0.92 ± 0.21	0.48 ± 0.09

Each ultrasound transducer was secured to a translational motor (VT-80 linear stage, Physik Instrumente, Auburn, MA) that was part of the stereotaxic imaging frame via a 3D-printed transducer holder (Fig. 1A). Ultrasound gel was applied directly to the surface of the brain, and the transducer was coupled to produce a coronal anatomical imaging plane. The motorized stage was then adjusted to find an imaging position that was at approximately bregma -2 mm. Care was taken to attempt to align each of the different transducers with the same anatomical imaging plane, using a combination of translational motor positions and qualitative assessment via ultrasound blood flow imaging (Fig. 1C).

For each dataset, a total acquisition time of 300 seconds (300,000 frames) of contrast-enhanced ultrasound data was acquired. Ultrasound data were stored as raw radiofrequency (RF) datasets for offline beamforming using the Verasonics pixel-based beamformer with a directional sensitivity of 0.8, corresponding to an F-number of approximately 1.5. Beamformed images were saved as in-phase/quadrature (IQ) data for further processing.

### 2.4 ULM reconstruction

All ULM reconstruction and analysis was performed in MATLAB (The MathWorks, Natick, MA; version R2022b). MB signal was extracted from the contrast-enhanced IQ data using singular value decomposition-based clutter filtering (Desailly et al., 2017), where the low-order tissue threshold was determined adaptively (Song et al., 2017b). This generally selected the first 20 singular values out of 1000 to be excluded. Filtering out of the higher order singular values, which is often done to suppress noise, was not performed as there was residual MB signal in the cortical regions of the brain. A noise-equalization profile was then applied (Song et al., 2017a), and an MB separation filter (Huang et al., 2020) was used to split the MB data into upward and downward components.

The filtered IQ data were then spatially interpolated to an isotropic λ/10 grid (L22-14vX: 9.8 μm; L35-16vX: 6.6 μm; MS-550S: 4.9 μm), and normalized cross-correlation with a threshold of 0.6 was performed with an empirically determined MB template (2D Gaussian) to produce sub-pixel localization candidates (Song et al., 2018). These candidates were then paired and tracked using the uTrack algorithm (Jaqaman et al., 2008) with a minimum persistence of 10 frames (10 ms). MB trajectories were then plotted onto a reconstruction grid space with a pixel size 2 μm and accumulated into a final super-resolved vascular image.

### 2.5 ULM image analysis

ULM image resolution was estimated using Fourier Ring Correlation (FRC) following the protocol outlined by (Hingot et al., 2021). Briefly, each MB track was randomly assigned to one of two ULM sub-accumulations, reconstructed with a pixel size of 1 μm, to produce independent reconstructions of the same vasculature. These two images were Fourier transformed, and the correlation between the two spectra was calculated for rings of increasing frequency to produce an FRC curve. This process was repeated in a bootstrapping protocol for five different random pairs of ULM sub-accumulations to get a more robust estimate of the FRC curve, with resolution estimation results reported as the mean value ± the standard deviation. The resolution of image was estimated using the two standards typically applied to ULM characterization: the ½-bit threshold and 2-σ criterion (van Heel & Schatz, 2005). The saturation rate of ULM images was quantified using an exponential saturation model as described by (Dencks et al., 2017; Lowerison et al., 2020), and the characteristic time (Hingot et al., 2019) of the ULM reconstruction was taken as the estimate to reach a 90% mapping of microvessels.

### 2.6 Transducer elevational beam width estimation

The elevational beam width of each transducer was estimated by scanning a zero-degree planewave transmit with a capsule hydrophone (HGL-0200, Onda Corporation, Sunnyvale, CA, USA) and ONDA AIMS III system (Onda Corporation) in a water tank. The ONDA position system was used to move the hydrophone in the axial direction starting from 2 mm away from the transducer surface to a maximum depth of 8 mm (0.05 mm increment) and from -2 mm to 2 mm in the elevational direction (0.02 mm increment). Elevational beamwidth was estimated as the full width at half max of the root mean square voltage at each axial position.

## 3 Results

### 3.1 MB PSF has reduced physical extent at higher frequency

**Fig. 2.**
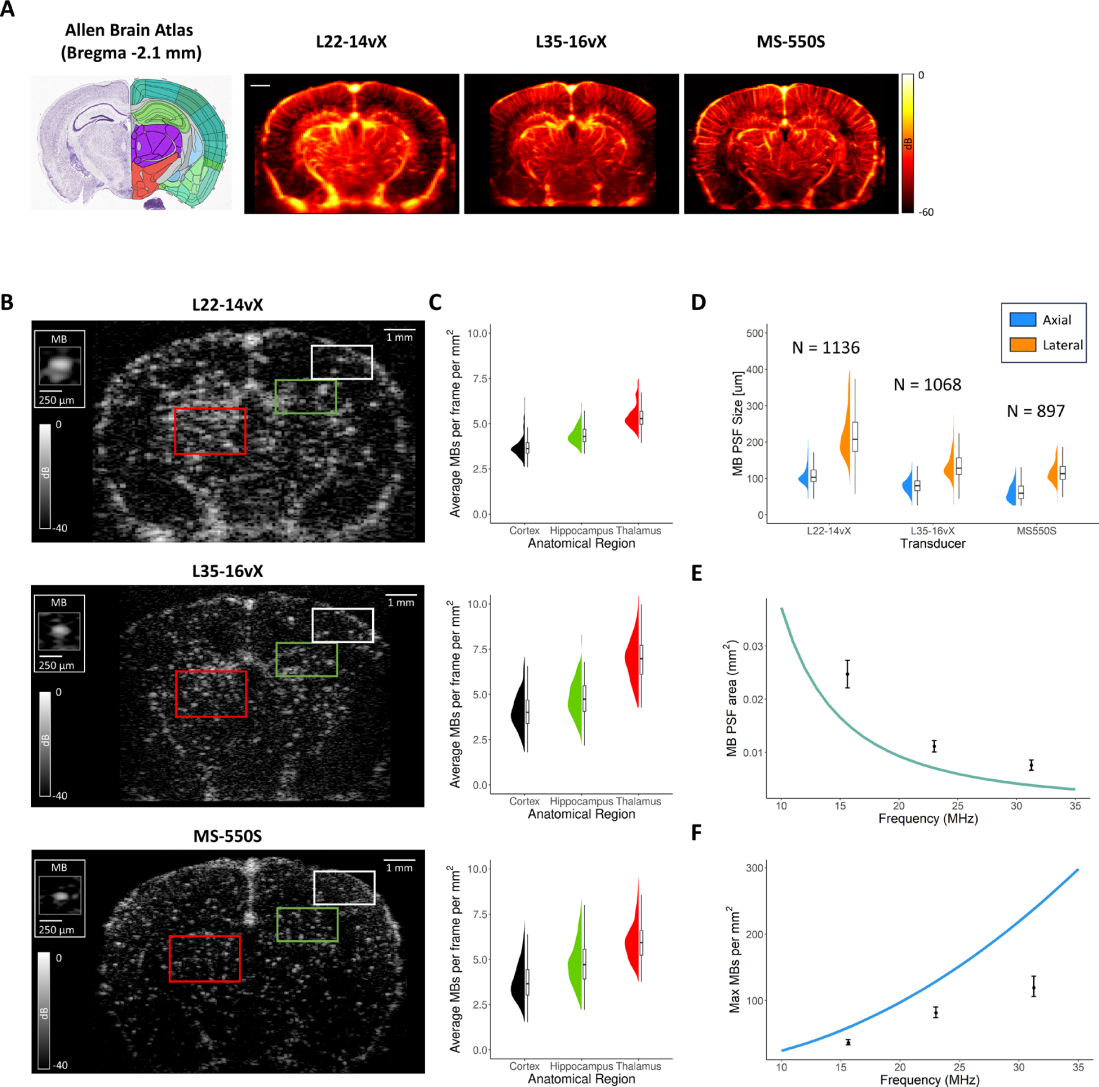
(A) Reference Allen Brain Atlas coronal section along with example of SVD clutter-filtered contrast-enhanced accumulations for the three different transducers. (B) The MB PSF has a reduced physical extent as the imaging frequency is increased, as shown by the figure insets demonstrating an isolated MB for each transducer. Three brain anatomies of interest are highlighted in the ultrasound images, the cortex (white box), the hippocampus (green box), and the thalamus (red box), which demonstrate (C) different local concentrations of MBs. There is substantial MB PSF overlap at lower frequency that is resolvable at higher frequency. (D) Experimental measurements of MB PSF sizes in both the axial and lateral dimensions for the three transducers. (E) A theoretical plot of MB PSF cross-sectional area with respect to imaging frequency, along with the experimental measurements for each transducer. (F) A hypothetical plot of the maximum number of resolvable MBs per square millimeter, with experimental estimates.

With the Allen Brain Atlas to provide anatomical context (Fig. 2A), example MB contrast-enhanced imaging frames for the three different transducers are demonstrated in Figure 2B, along with zoomed-in insets to identify individual isolated MBs. Three anatomical regions of interest are highlighted, corresponding to the cortex (white box), hippocampus (green box), and thalamus (red box). Based on this single animal experiment, we observed that the local concentration of MBs varies between these different regions, with the thalamus having a higher concentration than the cortex (Fig. 2C). However, it should be noted that this quantification depends on MB detection and localization criteria, which generally excludes overlapping MB signals and thus may underestimate MB counts under dense concentrations. As the imaging frequency is increased, the spatial extent of the MB PSF is reduced, potentially allowing for more efficient localization at higher MB concentrations. This is evident when comparing the thalamic region of the L22-14vX image, which has a substantial amount of MB PSF overlap in comparison to the MS-550S image, where the majority of MBs remain as isolated point targets.

In Figure 2D, we demonstrate experimentally determined MB PSF sizes, reported as the full width at half max in both the axial and lateral dimensions. For the L22-14vX, the MB PSFs were 113.0 ± 39.2 μm axial by 218.8 ± 66.3 μm lateral (number of isolated MBs analyzed N = 1136); for the L35-16vX, they were 80.9 ± 22.8 μm axial by 137.7 ± 47.4 μm lateral (N = 1068); and for the MS-550S, they were 63.7 ± 26.1 μm axial by 119.1 ± 36.8 μm lateral (N = 897). A rough approximation for the MB PSF cross-sectional area under these conditions is to consider it as an ellipse with an axial dimension equal to the wavelength and a lateral dimension of two wavelengths. This is demonstrated in the plot in Figure 2E, for frequencies ranging from 10 to 35 MHz, under the assumption of an average tissue sound speed of 1540 m/s. The experimentally determined MB PSF areas are included as points in this graph. To get an idea of how MB localization efficiency can improve with increasing frequency, we have plotted a theoretical “maximum” MB concentration in terms of resolvable MBs per millimeter squared of tissue, Figure 2F, estimated using the MB PSF area and the optimal packing density of elliptical surfaces (Chang & Wang, 2010).

### 3.2 Low MB concentration side-by-side comparison of ULM reconstruction

**Fig. 3.**
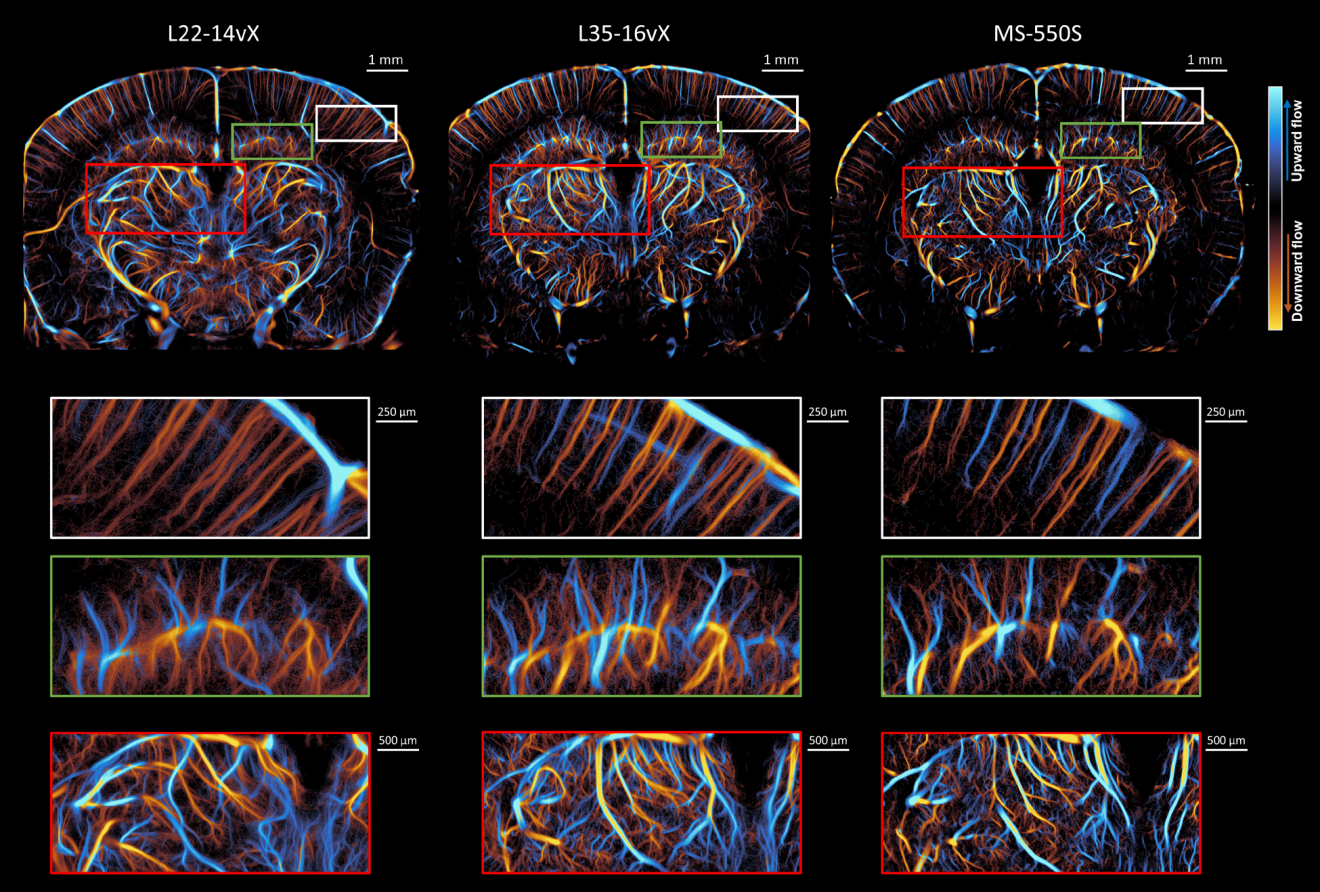
Side-by-side comparisons of the ULM reconstructions for the three different transducers at low MB concentration. The vessel saturation is decreased for increasing frequencies, especially for the deeper regions of the brain such as the entorhinal cortex. The white figure insets highlight the cortical vessel reconstruction, which demonstrated clearer vessel diameters at higher frequency. The green and red insets focus on the hippocampus and thalamus, respectively, where less noisy vascular reconstruction and more evident microvascular branching points are evident at high frequency.

An example of mouse brain ULM images with the L22-14vX, L35-16vX, and the MS-550S transducers is demonstrated in Figure 3, all taken under low MB concentration conditions that are optimized for the lower frequency transducer. We observe that increased frequency provided narrower vessel diameters in the cortex (white box) and clearer examples of microvascular branching points in the hippocampus (green box) and thalamus (red box), but caution should be taken when interpreting results which may come from misaligned regions of the brain. This difference is particularly evident in the thalamic region, which was previously noted to be a region with high local concentrations of MBs (Fig. 2B). However, we found that the ULM vessel saturation decreased with increased imaging frequency in the deepest regions of the brain (e.g., the entorhinal cortex). This can likely be attributed to a combination of narrower elevational beamwidth (Table 1), which reduces the sampling volume, and increased signal attenuation for higher frequencies.

**Fig. 4.**
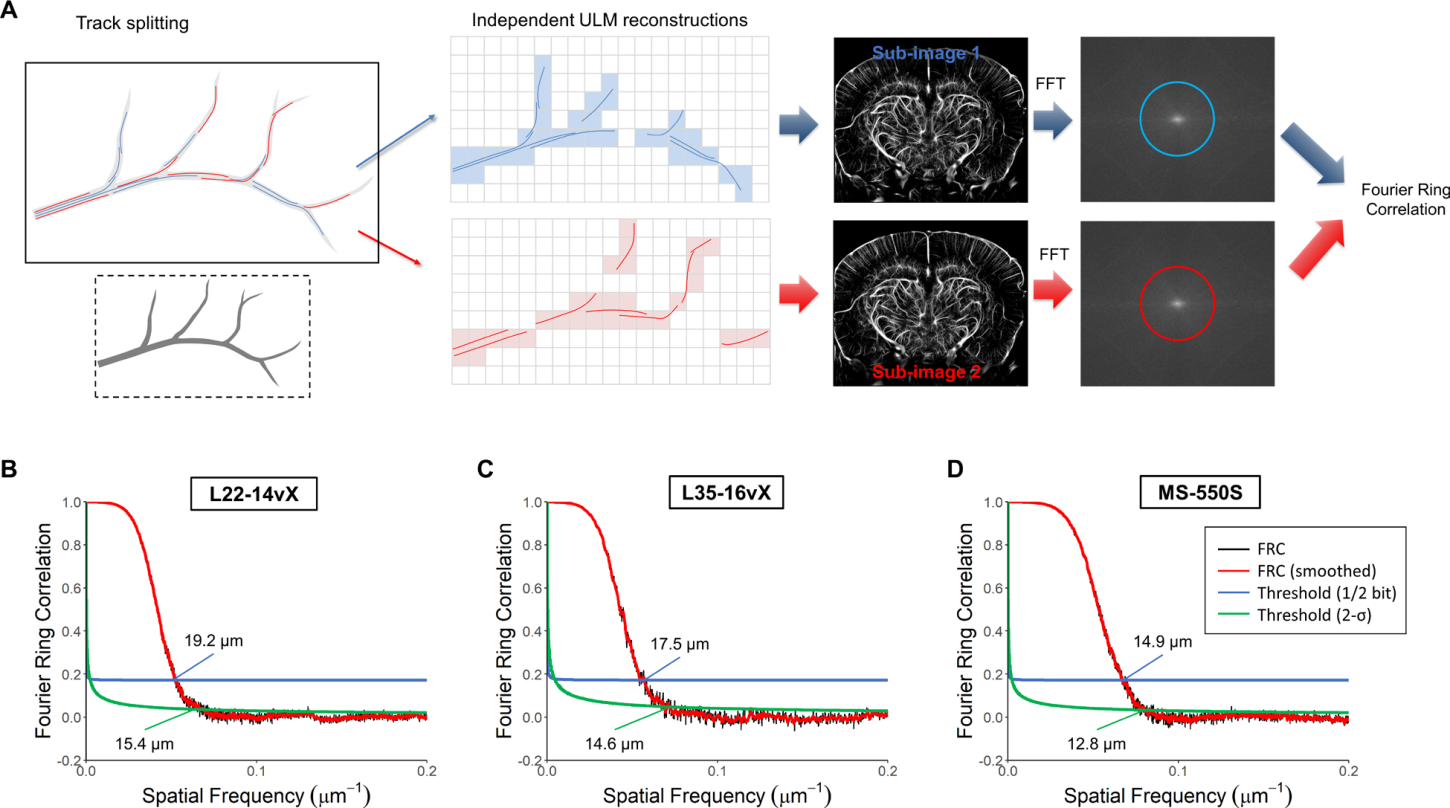
Fourier Ring Correlation resolution estimates for demonstrated low MB concentration datasets. (A) A diagrammatic example of the FRC process, where MB tracks are split into independent ULM reconstructions and compared. The FRC ½-bit and 2-σ estimates demonstrates a trend of gradually improved ULM resolution with increasing transducer frequency, with the (B) L22-14vX yielding a resolution estimate of 19.2/15.4 μm, the (C) L35-16vX yielding a resolution estimate of 17.5/14.6 μm, and the (D) MS-550S yielding a resolution estimate of 14.9/12.8 μm.

The estimated spatial resolutions for this dataset were quantified using Fourier Ring Correlation (Fig. 4). A diagrammatic example of the FRC process is demonstrated in Figure 4A, where individual MB tracks were accumulated into two independent ULM reconstructions. We found that there was a gradual improvement in the ULM resolution with increasing transducer frequency (Fig. 4B-D). The L22-14vX had a ½-bit resolution of 19.2 ± 0.3 μm; the L35-16vX had a ½-bit resolution of 17.5 ± 0.5 μm; and the MS-550S had a ½-bit resolution of 14.9 ± 0.2 μm. The 2-σ resolutions were 15.4 ± 0.8 μm, 14.6 ± 0.7 μm, and 12.8 ± 0.6 μm, respectively. It should be noted that the 2-σ threshold is close to the noise floor for ULM imaging, potentially explaining the higher standard deviation of FRC resolution estimates.

### 3.3 High MB concentration side-by-side comparison of ULM reconstruction

**Fig. 5.**
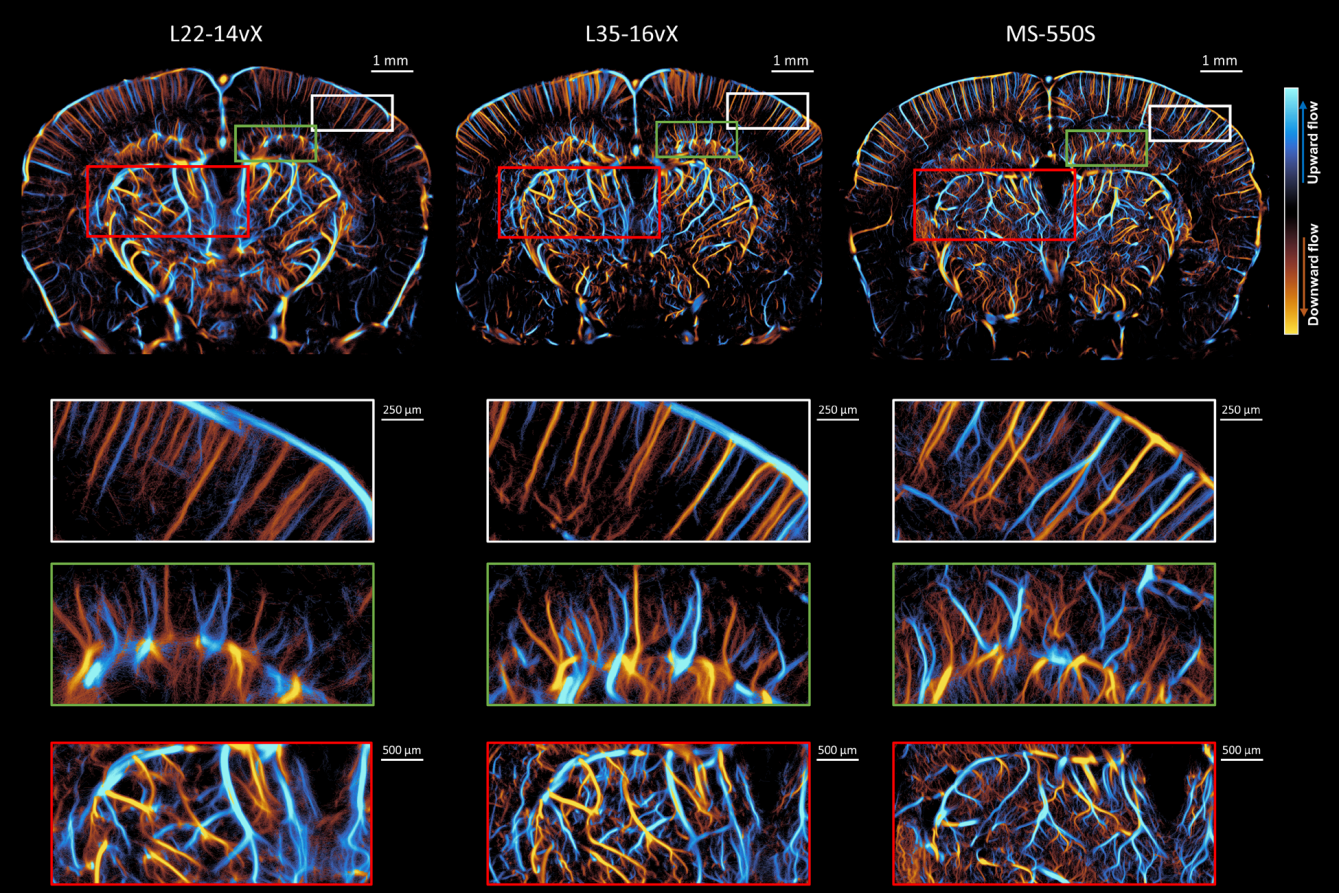
Side-by-side comparisons of the ULM reconstructions for the three different transducers at high MB concentration, with insets highlighting the cortex (white box), hippocampus (green box), and thalamus (red box). The L22-14vX demonstrates a degradation of ULM reconstruction quality, especially in the thalamic region (red box), where smaller vessels are difficult to visualize, and larger vessels are overemphasized. A similar observation is noted for the L35-16vX. The MS-550S had better saturation while maintaining clearly reconstructed vessels.

The same sort of comparison of mouse brain ULM images between the three transducers was also performed at a higher MB concentration (Fig. 5). The ULM image with the L22-14vX demonstrates a substantial blurring in vascular reconstruction, which is most pronounced in the thalamic region (red box). The microvascular lattice in this inset is noisy and difficult to visualize, and the larger vessels are overemphasized with indistinct branching points. A similar observation is noted for the L35-16vX in this region, although the degradation is less pronounced. The MS-550S does not exhibit this blurring vascular reconstruction, with better saturation under this higher concentration condition. The performance of the three transducers in the cortical region does not appear to be as impacted, likely due to the lower local concentration of MBs in the cortex noted in Figure 2.

**Fig. 6.**
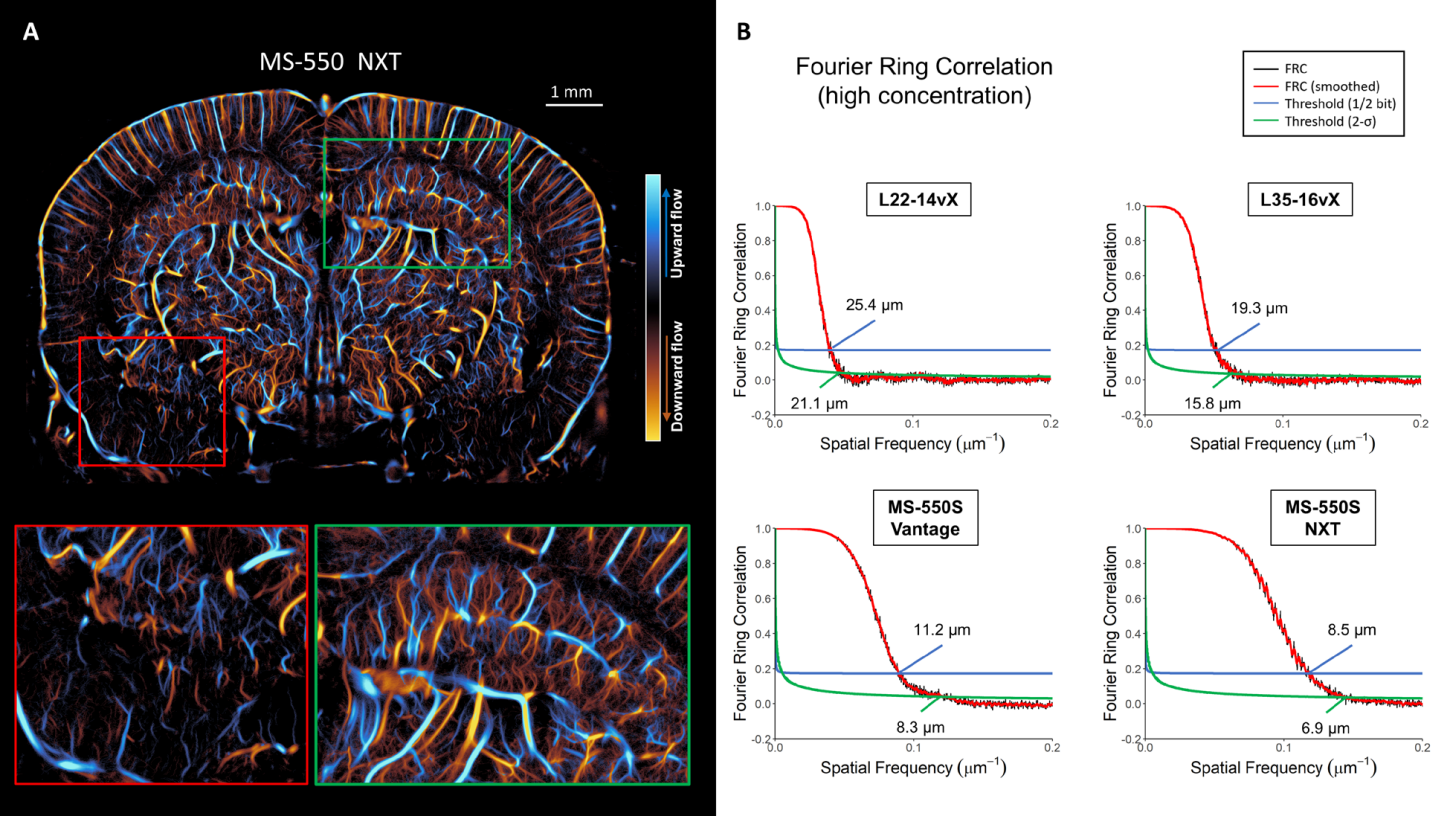
(A) High-frequency imaging was further tested on a Vantage NXT system. This resulted in a high degree of vessel saturation while maintaining reconstruction performance. The NXT had better performance for deeper brain regions, such as the entorhinal cortex (red box), than the Vantage 256. The hippocampal/white matter region (green box) also demonstrated better saturation with the NXT. (B) FRC resolution estimates for the high MB concentration datasets. The L22-14vX had a reduction in the resolution estimate in comparison to the low concentration case, with ½-bit and 2-σ estimates of 25.4 μm and 21.1 μm, respectively. The performance of the L35-16vX was slightly degraded in comparison to the lower concentration case at 19.3/15.8 μm. On the Vantage, the MS-550S resolution estimate was improved at higher MB concentration at 11.2/8.3 μm. An even finer resolution estimate was achieved with the NXT system, at 8.5/6.9 μm.

Another mouse was imaged using the MS-550S transducer at high MB concentration using the Verasonics NXT system. This is demonstrated in Figure 6A. We noted that the NXT was able to reconstruct more microvasculature in the entorhinal cortex (red box). The hippocampal/white matter region (green box) also demonstrated better saturation with the NXT. This is likely due to a combination of the NXT having a sampling rate that is better suited to imaging with the high-frequency MS-550S, and due to the increased acoustic output of the NXT relative to the Vantage 256.

**Fig. 7.**
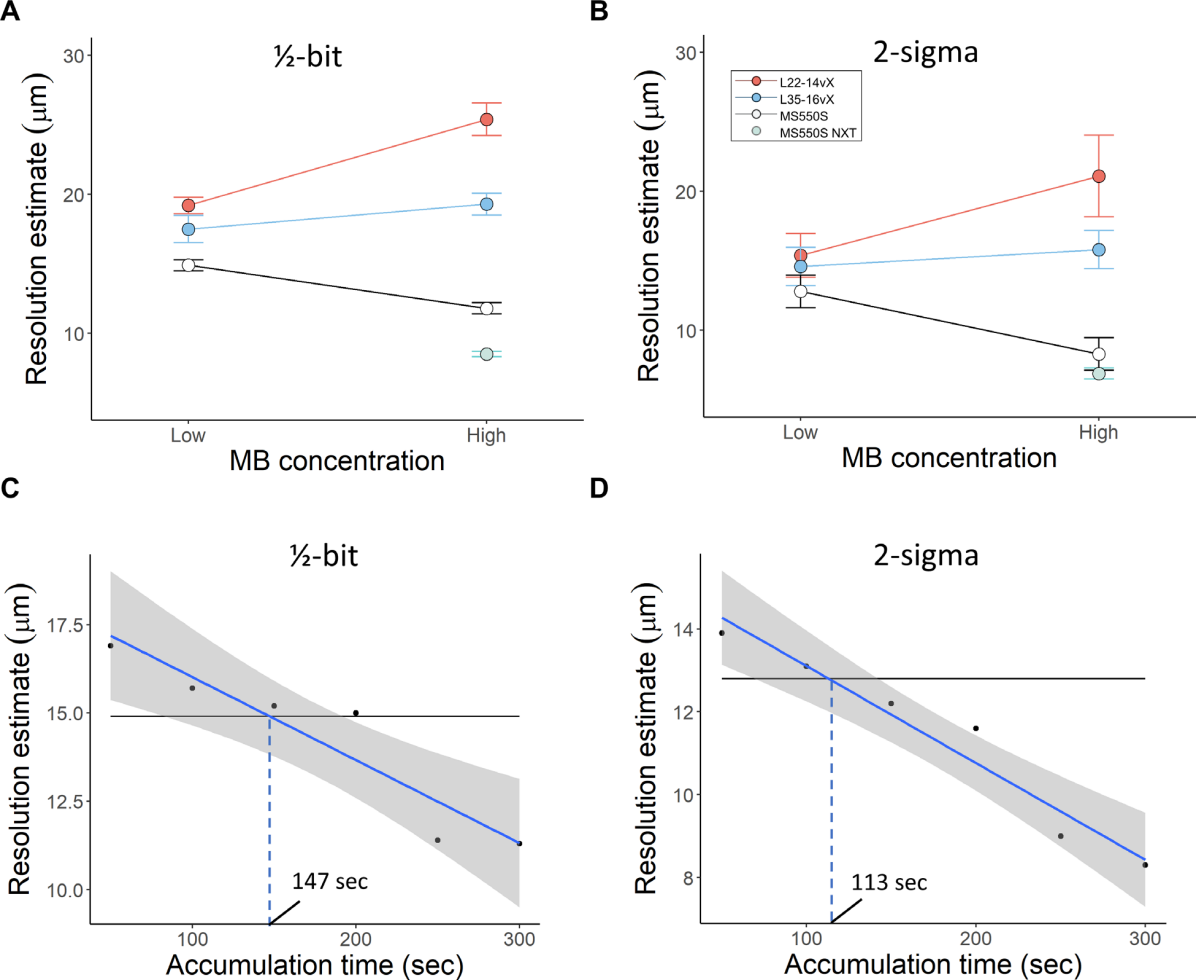
Summaries of the estimated FRC resolution are shown for (A) the ½-bit threshold and (B) the 2-σ threshold, with errors bars indicating the 95% confidence interval of the bootstrapping estimate. FRC estimates of gradual accumulations of high MB concentration data for the MS-550S probe to demonstrate the point at which it achieves the same resolution as the low MB concentration case, for (C) ½-bit threshold and (D) the 2-σ threshold.

FRC analysis was also performed on the high MB concentration datasets (Fig. 6B). The L22-14vX had a reduction in the resolution estimate in comparison to the low concentration case, with ½-bit and 2-σ estimates of 25.4 ± 0.6 μm and 21.1± 1.5 μm, respectively. The performance of the L35-16vX was slightly worse in comparison to the lower concentration case, with a resolution estimate of 19.3 ± 0.4 μm (½-bit threshold) and 15.8 ± 0.7 μm (2-σ threshold). We found that the Vantage MS-550S FRC analysis yielded a resolution estimate of 11.2 ± 0.2 μm (for ½-bit threshold) and 8.3 ± 0.6 μm (2-σ threshold). The NXT MS-550S FRC curve estimated a resolution of 8.5 ± 0.1 μm (for ½-bit threshold) and 6.9 ± 0.2 μm (2-σ threshold). These FRC quantifications are summarized in Figure 7A and 7B. The improvement in ULM resolution relative to low MB concentration datasets can likely be attributed to the increased vessel saturation, providing more instances of reproducible high-spatial frequency information.

### 3.4 High MB concentration reduces data accumulation time for high frequency dataset

An exponential saturation model was fit to a gradual accumulation of ULM acquisitions in order to estimate the 90% saturation time under each condition, that is, the point at which additional data accumulation would have a negligible impact on additional vascular information. We found that the L22-14vX had a saturation time of 181.7 seconds under low MB concentration and 209.8 seconds under high MB concentration. This slight increase in the saturation time may be indicative of noisy MB track generation arising from poor localization efficiency of overlapping MB signals. For the L35-16vX, we found that the saturation rate was slightly faster under high MB concentration (191.1 seconds for low MB concentration vs. 175.4 seconds for high MB concentration). For the MS-550S at low MB concentration, we found a saturation time of 307.2 seconds, implying that the MS-550S dataset was under accumulated in this scenario. For the high MB concentration case, the MS-550S saturation rate improved to 169.4 seconds. This was even more pronounced for the MS-550S data set acquired with the NXT system, which had a saturation time of 153.9 seconds.

The ULM saturation rate also needs to be considered within the context of the achieved resolution, as the L22-14vX and L35-16vX had impaired localization efficacy at higher MB concentration, despite similar saturation rates under both conditions. To test the hypothesis that high MB concentration would reduce data accumulation times, we generated gradual sub-accumulations of high MB concentration MS-550S data in order to estimate the point at which it achieved the same FRC resolution as the low MB concentration MS-550S case. This is demonstrated in Figure 7C for the ½-bit threshold, which estimated that we would need 147 seconds of high MB concentration data to achieve the low MB concentration MS-550S performance, and in Figure 7D for the 2-σ threshold, which predicted only 113 seconds of ULM data.

## 4 Discussion/Conclusion

The preeminent accomplishment of a cerebrovascular imaging technology would be the successful mapping of the microvasculature with a high degree of confidence and fidelity throughout the entire depth of the brain. ULM side-steps the conventional trade-off between resolution and penetration depth in ultrasound: the localization process substantially improves resolution without sacrificing the imaging field-of-view (Couture et al., 2018). Under the assumption of perfect imaging conditions (e.g., no tissue motion, no aberration, no MB signal overlap/interference, sufficient signal-to-noise ratio), the ideal ULM localization accuracy is determined by the Cramér-Rao lower bound (Desailly et al., 2015), which predicts a sub-capillary resolution even at clinical imaging frequencies. Empirically, the *in vivo* localization accuracies reported in the literature tend to be limited to one-tenth of the wavelength (Desailly et al., 2013; Hingot et al., 2021), arising from non-ideal imaging conditions such as high tissue motion and imperfect speed-of-sound estimation. This empirical limit should be sufficiently precise to resolve capillary-scale microvessels for small animal imaging (typically done at imaging frequencies in the range of 15 MHz); however the relative impact of tissue motion on ULM resolution will be more dominant at these higher imaging frequencies. Furthermore, the stochastic nature of ULM makes MB traversal for a specific microvessel incredibly rare (Christensen-Jeffries et al., 2019; Hingot et al., 2019; Lowerison et al., 2020), reducing the probability and confidence for microvascular imaging with ULM. The combination of the pursuit of small vessel mapping and the stochastic nature of ULM also exacerbates the requirement for prohibitively long data acquisition times.

In this report, we investigated ULM reconstruction of mouse brain imaging for three ultrasound imaging transducers with different imaging frequencies to test the hypothesis that increased frequency will allow for reduced data acquisition times. We found that increasing the imaging frequency decreased the spatial extent of the MB PSF (Fig. 2), enabling localization at high(er) MB concentrations. This observation is supported by literature which models the number of MBs that can occupy the same unit volume while remaining localizable (Belgharbi et al., 2023; Christensen-Jeffries et al., 2019). This was particularly relevant for accurate reconstruction of the thalamic regions of the brain, which tended to have higher local concentrations of MBs in comparison to the cortex (Fig. 2B & C). Interestingly, a substantial difference in blood volume between these regions was not noticed in the power Doppler images (Fig. 2A), nor is it expected that the cortex would have reduced vascularization in comparison to the thalamus (Di Giovanna et al., 2018). We, therefore, speculate that this difference in regional MB concentration may be due to the impact of anesthesia (Wang et al., 2025) or the open craniotomy procedure, both of which may influence the cerebral blood flow. One should note that the experimental PSF sizes were larger than the theoretical wavelength for the transducer center frequencies (i.e., 98.6 μm at 15.625MHz for the L22-14vX; 66.9 μm at 23MHz for the L35-16vX; and 49 μm at 31.25MHz for the MS-550S), which can be attributed to factors such as diffraction, low number of compounding angles, and sound-speed inhomogeneities. It should also be noted that the backscattered signal intensity of MBs rapidly decreases for frequencies > 7 MHz (Gorce et al., 2000) which, in conjunction with the frequency-dependent attenuation of ultrasound signal, could lead to an effectively “sparser” distribution of MBs at higher frequencies independent of the decrease in PSF size.

For the transducer comparison under the same low MB concentration experimental conditions, we found that the highest frequency probe yielded ULM images with narrower vessel diameters in the cortex, and clearer reconstruction of the microvasculature in the hippocampus and thalamus, in comparison to the lower frequency probes. However, the ULM vessel saturation was reduced for the high-frequency probe, which is likely the result of the narrower elevational beamwidth (Table 1). This smaller sampling volume can be beneficial for MB localization at higher concentrations: two-dimensional ULM suffers from projection of three-dimensional vasculature (Heiles et al., 2022), leading to overlapping PSFs from distant MBs and elevational ambiguity of MB trajectories. A narrower elevational beamwidth reduces the probability that MBs will overlap, leading to more efficient localization and better elevational resolution. This elevational ambiguity is also problematic for the MB tracking step, as physically distant MBs can be erroneously paired, which can potentially obscure microvascular flow. It is worth noting that previous research has demonstrated that these out-of-plane MB positions can be useful for elevational estimation/visualization in 2D ULM (Renaudin et al., 2023), but this was unexplored in this report.

Under the high(er) MB concentration condition, we found that ULM performance of the lowest frequency probe was substantially degraded, especially in the thalamic regions of the brain: the microvascular bed was indistinct/obscure, and the larger vessels lacked obvious branching points. The L35-16vX was less impacted, however the major vessels appear to be overemphasized relative to the low MB concentration reconstruction. Notably, these regions still have a blurry “vessel-like” appearance, with some noisy background haze, which may stem from false localizations of MB interference patterns. These interference patterns will tend to create local maxima that are situated close to the vessel lumen, but which lack the precision and consistency of true MB point localizations. In comparison, the ULM performance was improved for the highest frequency probe, with an increase in the overall saturation, especially for the dataset acquired with the NXT system.

The FRC analysis demonstrated that the estimate for the ULM reconstruction resolution modestly improved with increasing frequency under the low MB concentration condition, from 15.4 ± 0.8 μm at the lowest frequency up to 12.8 ± 0.6 μm at the highest frequency. For the higher MB concentration datasets, we found that the lowest frequency probe had a worse resolution estimate (21.1± 1.5 μm) and a marginal decrease in performance for the mid-frequency probe (15.8 ± 0.7 μm). For the highest frequency probe, the MS-550S transducer, the FRC resolution estimate improved drastically to 8.3 ± 0.6 μm (Vantage 256) and 6.9 ± 0.2 μm (NXT). This substantial improvement in the high concentration MS-550S result in comparison to the low concentration MS-550S result is probably attributable to the increase in vessel saturation. FRC is ultimately a measure of consistency between the two independent ULM reconstructions. For the low concentration MS-550S ULM reconstruction, there are likely microvascular MB trajectories which are only present in one of the two sub-images used for FRC analysis. This would lead to these trajectories being discounted as reconstruction noise on the FRC curve. With the higher MB concentration data, there are more overall microvascular trajectories being accumulated, increasing the consistency of the FRC sub-images, and thus improving the quantified spatial resolution. This provides evidence to support our initial hypothesis that increasing the imaging frequency provides an avenue for enabling ULM at high(er) concentrations of MBs, and thus reduces the total accumulation time required for microvascular reconstruction. Expressed as a fraction of the wavelength, the estimated FRC resolutions were 0.16λ, 0.21λ, and 0.26λ in the low MB concentration case for the L22-14vX, L35-16vX, and MS-550S, respectively. For the high MB concentration case, they were 0.21λ, 0.23λ, and 0.17λ, respectively, with the NXT MS-550S acquisition achieving 0.14λ. In all cases, these performed worse than the empirical one-tenth of a wavelength which is often mentioned in literature. We also estimated the reduction in data accumulation time using ULM saturation curves and FRC estimates from gradual accumulations of MS-550S ULM data. In both cases, the required acquisition time was roughly half for the MS-550S, which is in line with the difference in MB concentration between the low and high MB conditions.

There are several limitations which should also be discussed. For this report we limited our discussion to neuroimaging in small animal models, where the imaging depth is shallow and imaging frequency is usually >15 MHz; thus, the empirical limit of one-tenth of the wavelength (Desailly et al., 2013; Hingot et al., 2021) should be sufficiently precise to resolve microvessels. It would be challenging to observe these microvascular flow events in larger animals and in humans. High-frequency imaging has higher signal attenuation, which limits MB detection in deep regions. Increasing the transmit power can help in this scenario; however, there is a risk of more native red blood cell backscatter in the near field (Kuo & Shung, 1994), which can lead to incorrect localizations in the shallow regions of the brain. Higher frequency imaging also requires a higher data sampling rate, which can increase the raw data size and computational requirements. ULM reconstructions and therefore estimates of ULM resolution were performed on datasets without motion compensation. It should be noted that tissue motion can have a substantial impact on ULM performance, motivating several investigations into methods for correcting for this motion (Harput et al., 2018; Hingot et al., 2017). This is particularly relevant at higher frequencies, where tissue motion may become the dominant factor in super-localization. This study is also lacking external validation techniques, such as multiphoton microscopy or histology verification, for the differences in microvascular detail between the difference transducer frequencies, instead relying on internal measures of ULM consistency (i.e., FRC). Such external validation methods are not trivial to perform, due to differences in sampling volumes and challenges with multimodal registration and should be the subject of future research.

The discussion of the ultrasound PSF with respect to the imaging frequency was simplified for brevity/clarity in the initial hypothesis. Ultrasound has an anisotropic and spatially variant PSF. For planewave compounding the axial imaging resolution depends on the center frequency, bandwidth, and number of cycles of the transmit pulse. The lateral resolution depends on the size of the lateral aperture and the extent of the tilting angles. Although some of these parameters were kept consistent (i.e., transmit pulse cycles and tilting angles), others, such as the lateral aperture, were not investigated. It is worth noting that the MS-550S has a larger lateral aperture (14.08 mm) than either the L22-14vX (12.8 mm) or the L35-16vX (8.9 mm), which likely impacted the ULM reconstruction fidelity beyond the change in frequency. Likewise, the elevational beamwidth of each transducer is different, which alters the sampling volume and thus efficiency of MB localization.

ULM imaging provides a rich dataset of macro- and micro-vascular detail, potentially allowing for a plethora of information for semi-automated vascular digitalization strategies (Strumane et al., 2025). Such processing methods can enable in-depth quantification of vessel lengths, diameters, and tortuosity on both a global and a local scale (Wang et al., 2025). However, it should be noted that vessel reconstruction under ULM, which relies on the accumulation of MB trajectory information, is not continuous and thus needs to be interpreted with caution. There has been substantial effort in developing MB track clustering methods for quantification of vascular features in 3D ULM (Heiles et al., 2022), but to our knowledge such strategies have not been applied robustly to 2D ULM. This is an ongoing and active area of research.

ULM breaks the diffraction limit via localization of MBs, side-stepping the classic ultrasound trade-off between imaging resolution and penetration depth. Instead, the trade-off is between spatial resolution and data acquisition time. In this report, we found that increasing ultrasound frequency can still play a part in this milieu by enabling localization of higher MB concentrations, thus improving vessel saturation rate and confidence in microvascular reconstruction for neuroimaging research. This process is straightforward and accessible in the pre-clinical imaging space due to the prevalence of commercial high-frequency imaging probes.

## Data Availability

The data supporting the findings in the publication are available upon reasonable request.
